# Association of Early Heparin Use with Mortality in Cirrhotic Patients: A Retrospective Cohort Study

**DOI:** 10.5152/tjg.2026.25297

**Published:** 2026-03-10

**Authors:** Maodong Guo, Tao Ling, Jin Ding, Xin Shi

**Affiliations:** Department of Gastroenterology, Jinhua Hospital of Zhejiang University, Jinhua, Zhejiang, P.R. China

**Keywords:** Anticoagulation, heparin, liver cirrhosis, mortality

## Abstract

**Background/Aims:**

Controversy remains regarding anticoagulant use to improve the prognosis of cirrhosis. Herein, we evaluated the effect of early use of unfractionated heparin (UFH) on survival in cirrhotic patients.

**Materials and Methods:**

Data were acquired from the Medical Information Mart for Intensive Care-IV database. Patients were allocated into early heparin and control groups according to the presence of UFH treatment within 48 hours following intensive care unit admissions. Effects of early UFH use on survival in cirrhotic patients were assessed using Kaplan–Meier survival curves with the log-rank test and Cox proportional hazards model. Associations between UFH use and the risk of red blood cell (RBC) transfusion were determined with the logistic regression model. The robustness of the results was assured with propensity score matching (PSM).

**Results:**

This study included 1652 cirrhotic patients (781 and 871 in the early heparin and control groups, respectively). At 30-day follow-up, mortality was prominently lower in the early heparin group than in the control group (*P* < .001). Furthermore, early heparin use was associated with diminished 30-day mortality (hazards ratio [HR]: 0.742; 95% CI: 0.599-0.919; *P* = .006). This result was robust even after PSM (HR: 0.729; 95% CI: 0.568-0.937; *P* = .014). UFH use was not strikingly associated with an augmented risk of transfusion before (odds ratio [OR]: 0.917; 95% CI: 0.693-1.215; *P* = .547) and after (OR: 0.857; 95% CI: 0.616-1.191; *P* = .357) PSM.

**Conclusion:**

Early heparin use was associated with reduced 30-day mortality among cirrhotic patients, without increasing the risk of RBC transfusion.

**Main Points**

- Patients with liver cirrhosis are at higher risk of thrombotic complications, which have various effects on the course of the disease.
- There are still limited data and no clear consensus guidelines support anticoagulants for cirrhosis treatment, which calls for further investigation.
- Early heparin use was associated with reduction in 30-day mortality in the intensive care unit patients with cirrhosis, without a concomitant increase in the risk of red blood cell transfusion.

## Introduction

Coagulation imbalance is an essential complication of cirrhosis, which has various effects on the course of cirrhosis.^1^ Recently, cirrhosis has been considered a hypercoagulable disease, instead of a hypocoagulable disease, due to the high incidence of thrombotic complications in cirrhotic patients.^2^^,^^3^ In fact, recent literature has revealed a prevalence rate of thrombotic events ranging from 0.5% to 6.3% in hospitalized cirrhotic patients,^4^ which is 1.7-fold higher than that in the general population.^5^ Additionally, portal vein thrombosis (PVT), although its incidence is estimated to be <1.0% in the general population, has been shown to affect 5%-10% of cirrhotic patients annually.^6^^,^^7^ A prior study demonstrated that venous thrombosis secondary to a hypercoagulable disorder was associated with advanced cirrhosis progression.^8^ Through a retrospective study, Søgaard noted a higher 30-day mortality rate in patients with venous thromboembolism (VTE) combined with cirrhosis than in patients without cirrhosis.^9^ Also, a recent meta-analysis by Xian revealed that PVT elevated hepatic decompensation events, thus affecting the short-term prognosis of cirrhotic patients.^10^ Mounting animal studies have strongly evidenced that coagulation cascades can directly favor the development of liver fibrosis, while anticoagulant administration improved both portal hypertension and liver fibrosis in cirrhotic animal models,^11^^,^^12^ highlighting anticoagulant treatment as a novel potential approach to delay hepatic decompensation. However, there are still limited data and no clear consensus guidelines support anticoagulants for cirrhosis treatment, which calls for further investigation.

As a polydisperse mixture of glycosaminoglycans, unfractionated heparin (UFH) has been broadly utilized as an anticoagulation treatment to prevent venous thromboembolism because of its strong anticoagulant effects. Unfractionated heparin is preferred for critically ill patients in intensive care units since it has a relatively quick onset and offset with almost completely reversible effects.^13^ Accumulating clinical evidence has demonstrated that early UFH treatment markedly elevated the survival of several hypercoagulation-associated diseases, such as sepsis and malignant tumors.^14^^,^^15^ Nevertheless, it remains unclear whether UFH treatment can improve survival in cirrhotic patients. Therefore, this study probed the effect of early UFH use on survival in cirrhotic patients by retrospectively analyzing data from the Medical Information Mart for Intensive Care-IV (MIMIC-IV) database.

## Materials and Methods

### Database Introduction

With pgAdmin4 PostgreSQL 9.6 and structured query language, data were retrieved from MIMIC-IV, which is a large and publicly accessible database that offers comprehensive critical care data of patients admitted to the Beth Israel Deaconess Medical Center (BIDMC) in Boston from 2008 to 2019. The Institutional Review Board of BIDMC granted a consent waiver and approved research resource sharing. In addition, this study was exempt from formal ethical approval by the Institutional Review Board of Jinhua Hospital of Zhejiang University (Waiver Number: MC2025-04; Date: May 20, 2025). The Collaborative Institutional Training Initiative examination was completed, and a corresponding certificate for the data used was acquired.

### Inclusion and Exclusion Criteria

**Figure 1.**
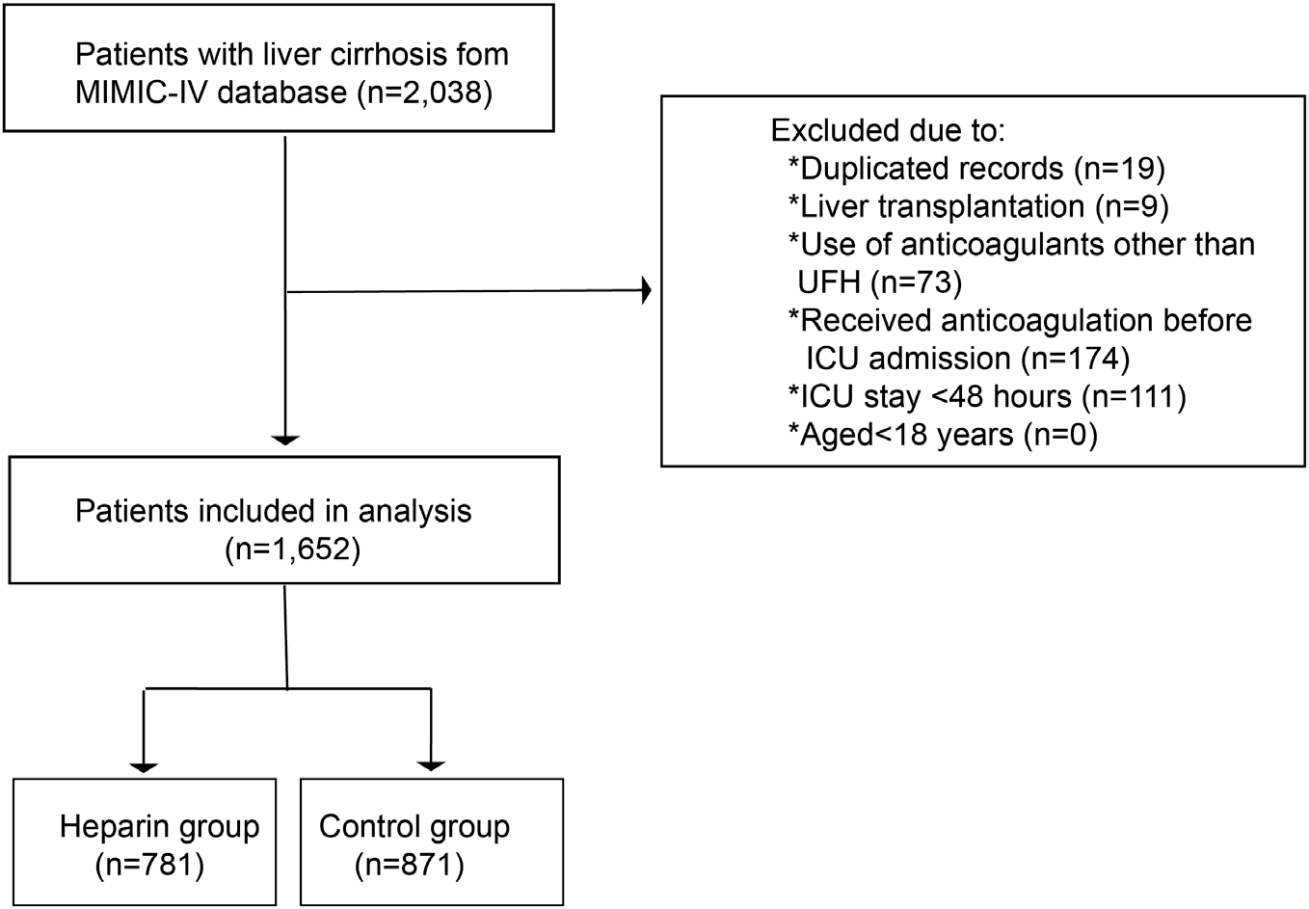
Flow chart of the study.

The present study focused on critically ill patients with cirrhosis. Cirrhosis was diagnosed based on the International Classification of Diseases (9th and 10th editions). The following exclusion criteria were utilized: (1) individuals undergoing liver transplantation; (2) individuals of ≤ 18 years; (3) individuals staying in intensive care units (ICUs) for <48 hours; (4) individuals using other anticoagulants (low-molecular-weight heparin (LMWH), warfarin, rivaroxaban, argatroban, and bivalirudin) but not UFH; (5) patients receiving any anticoagulation treatments prior to ICU admissions. For patients with multiple ICU admissions, only the initial ICU admission data from their first hospitalization were obtained. The workflow of the study is detailed in Figure 1.

### Data Collection

Data extracted from the database included many parameters, such as baseline characteristics, characteristics (age and sex), disease severity scores, etiology of cirrhosis, comorbidities and complications, laboratory test results, and details of clinical therapies. Disease severity was evaluated with sequential organ failure assessment (SOFA) and model for end-stage liver disease (MELD) scores. Comorbidities and complications included heart failure, renal failure, diabetes mellitus, chronic pulmonary disease, sepsis, VTE, hepatic encephalopathy (HE), PVT, liver cancer, upper gastrointestinal bleeding (UGIB), and variceal bleeding. Laboratory test results included sodium, chloride, hemoglobin (HB), calcium, magnesium, phosphorus, white blood cell (WBC) count, alanine transaminase, platelet, albumin, aspartate transaminase, bilirubin, international normalized ratio (INR), partial thromboplastin time, and serum creatinine. Laboratory test results were the initial values within 48 hours following ICU admissions. Clinical therapies within 48 hours following ICU admissions consisted of mechanical ventilation and drug (albumin, diuretics, vasopressor, and UFH) administration. Red blood cell (RBC) transfusion was assessed 48 hours after ICU admissions.

Patients who received UFH within 48 hours following ICU admissions were classified as the early heparin group, whereas who did not receive UFH within 48 hours following ICU admissions were allocated as the control group. The dosages of UFH used within 48 hours following ICU admissions were also recorded. The primary outcome was 30-day mortality, and the secondary outcome was the incidence of red blood cell transfusion within 48 hours of ICU admission.

### Statistical Analysis

Variables with missing values >15% were excluded to minimize bias. For variables with missing values <5%, the missing values were substituted with the median or mean of the variable. For variables with missing values >5%, the missing values were imputed with the multiple imputation method. Continuous variables with skewed distribution were described as the mean (SD) or median (interquartile ranges). Intergroup comparisons were carried out with independent *t* (data with normal distribution), Pearson chi-squared (categorical variables), and Mann–Whitney (skewed distribution) tests, as appropriate. Survival curves were plotted with the Kaplan–Meier method and compared with the log-rank test. Associations between UFH use, the dosages of UFH, and 30-day mortality were determined with Cox regression. Due to sample size limitations, this study categorized patients into 2 groups based on UFH dosage: the ≤5000 IU group and the >5000 IU group, to investigate the impact of different UFH doses on mortality in cirrhotic patients. Adjusted covariates were chosen as per *P* < .05 in the univariate analysis and relevant clinical experience. The impact of UFH use on RBC transfusion was identified with logistic regression. MELD scores (≤20, >20), SOFA scores (<8, ≥8), sepsis, PVT, and HE were subjected to stratified and interaction analyses. The robustness of the findings was assured by balancing potential confounders with nearest-neighbor propensity score matching (PSM). The R statistical package version 3.6.3 (R Foundation for Statistical Computing; Vienna, Austria) was employed for all analyses, and 2-tailed *P* < .05 represented a statistically significant difference.

## Results

### Characteristics of the Included Patients

Based on the inclusion and exclusion criteria, a total of 1,652 patients were ultimately included in the detailed data analysis, of whom 781 (47.28%) patients received early UFH treatment. Table 1 displays various basic characteristics of patients with UFH use and controls, which revealed prominent differences between the 2 groups. Specifically, patients who received early UFH treatment were older (*P* = .012), and more likely to receive albumin therapy (*P* = .010), with lower incidence rates of alcoholic cirrhosis (*P* < .001), HE (*P* = .017), UGIB (*P* < .001), and variceal bleeding (*P* < .001). The early heparin group also had lower levels of albumin (*P* = .027), HB (*P* < .001), sodium (*P* < .001), chloride (*P* = .012), bilirubin (*P* < .001), and INR (*P* < .001) but higher levels of calcium (*P* = .006), platelets (*P* < .001), and WBC count (*P* = .003). Meanwhile, greatly lower MELD (*P* = .006) and SOFA scores (*P* = .037) were observed in the early heparin group compared with the control group.

### Propensity Score–Matched Cohorts

**Figure 2.**
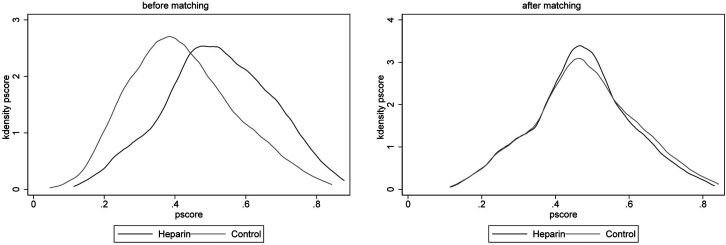
Kernel density plots of the propensity scores before and after propensity score matching.

To minimize the effect of potential confounders, 1 : 1 nearest-neighbor PSM was carried out with a caliper width of 0.02. Through logistic regression models, propensity scores were calculated with imbalanced covariates, including age, albumin, platelet, sodium, HB, chloride, bilirubin, INR, calcium, WBC, alcoholic cirrhosis, HE, MELD scores, SOFA scores, and albumin use. Thereafter, the degree of PSM was determined with the kernel density plot of *P*. Subsequent to PSM, 556 pairs of participants were well matched, and baseline characteristics were balanced between the 2 groups (all *P* < .05, Table 2, Figure 2).

### Survival Analysis

**Figure 3.**
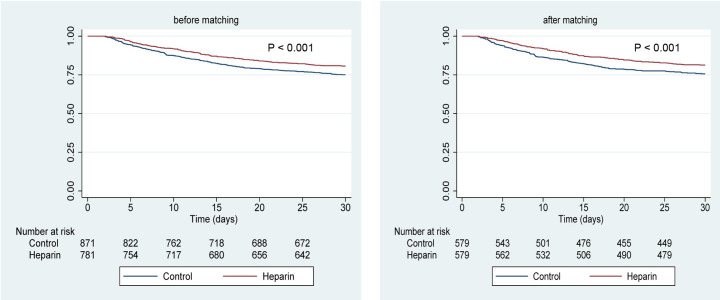
Kaplan–Meier survival curves of unfractionated heparin use and 30-day mortality.

The 30-day mortality rates were 19.33% and 24.91% in the early heparin and control groups, respectively. Firstly, the probability of 30-day survival in the 2 groups was analyzed with Kaplan–Meier survival curves. The data (Figure 3) revealed that 30-day survival rates were substantially higher among patients receiving early UFH treatment than among controls, both before and after PSM (the log-rank test: *P* < .001). Then, the association of UFH use with 30-day mortality was clarified with 4 Cox proportional hazards models (Table 3). In the pre-matched cohort, UFH use exhibited an association with diminished during 30-day follow-up in model I (without adjustment; hazard ratio [HR]: 0.744; 95% CI: 0.605-0.917; *P* = .005), model II (with adjustment of age, MELD scores, and SOFA scores; HR: 0.770; 95% CI: 0.626-0.949; *P* = .014), model III (with adjustment of age, MELD scores, SOFA scores, INR, albumin, albumin use, and sodium; HR: 0.753; 95% CI: 0.609-0.930; *P* = .008), and model IV (with adjustment of age, MELD scores, SOFA scores, INR, albumin, albumin use, sodium, alcoholic cirrhosis, chloride, calcium, HB, platelet, WBC, bilirubin, HE, and mechanical ventilation; HR: 0.742; 95% CI: 0.599-0.919; *P* = .006). Similarly, in the matched cohort, UFH use was also associated with augmented 30-day survival rates (HR: 0.729; 95% CI: 0.568-0.937; *P* = .014) (Table 3), validating the robustness of the results.

**Figure 4.**
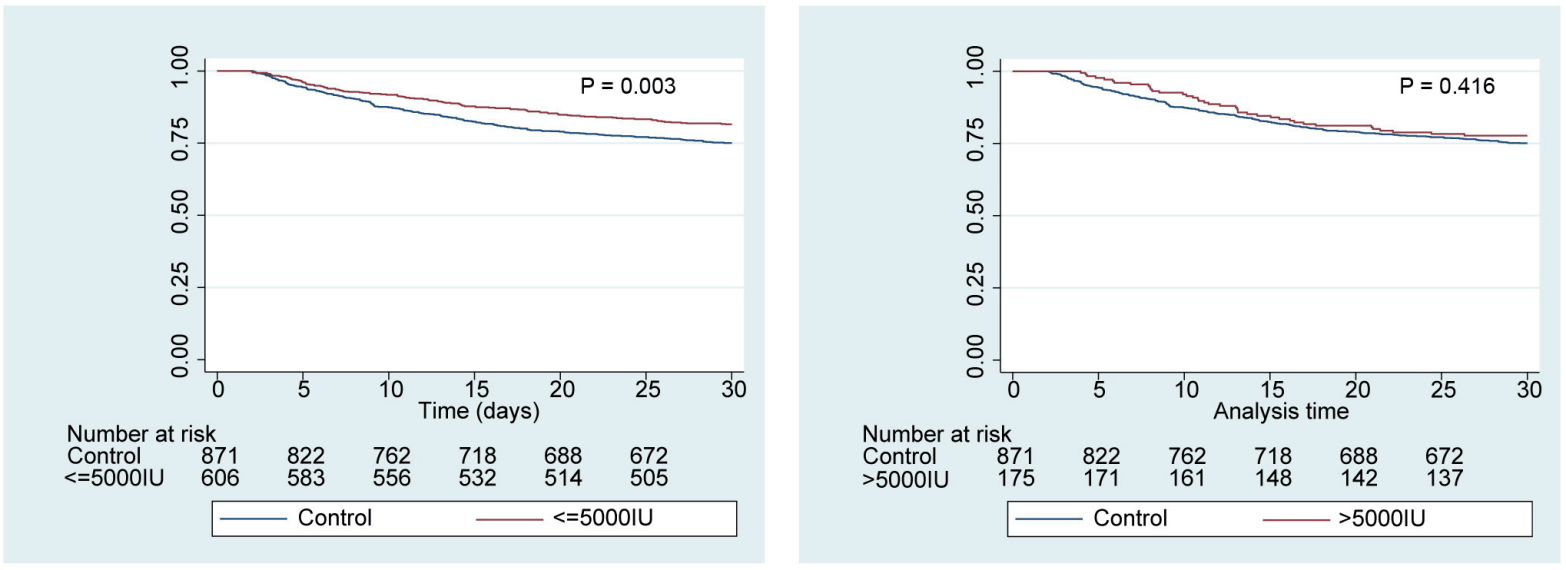
Kaplan–Meier survival curves of different dosages of unfractionated heparin use and 30-day mortality.

Kaplan–Meier survival curve analysis of the 30-day survival probabilities for the different dosages of UFH and outcomes showed that (Figure 4), compared to the control group, the 30-day survival rate for patients in the ≤5000 IU group was significantly higher (log-rank test: *P* = .003). Although the >5000 IU group also had a higher 30-day survival rate compared to the control group, the difference did not reach statistical significance (log-rank test: *P* = .416). Similar results were confirmed in the Cox regression analysis (Table 4). Compared to the control group, patients in the ≤5000 IU group demonstrated a significantly higher 30-day survival rate (HR: 0.766; 95% CI: 0.608-0.965; *P* = .024). In contrast, while the 30-day survival rate for the >5000 IU group was higher than that of the control group, the difference did not achieve statistical significance (HR: 0.712; 95% CI: 0.498-1.016; *P* = .061).

### Relationship Between Heparin Use and the Secondary Outcomes

Logistic regression analyses demonstrated that UFH use that UFH use was not conspicuously associated with an augmented risk of transfusion either before (odds ratio [OR]: 0.917; 95% CI: 0.693-1.215; *P* = .547) or after (OR: 0.857; 95% CI: 0.616-1.191; *P* = .357) PSM.

### Stratified and Interaction Analyses

The relationship between UFH therapy and 30-day mortality in various subgroups is presented in Table 5. Unfractionated heparin use had significant HR in several subgroups, including MELD score > 20 (HR: 0.660; 95% CI: 0.505-0.862; *P* = .002), SOFA score ≥ 8 (HR: 0.667; 95% CI: 0.507-0.878; *P* = .004), presence of sepsis (HR: 0.675; 95% CI: 0.522-0.874; *P* = .003), presence or absence of HE (HR: 0.509; 95% CI: 0.263-0.983; *P* = .044; HR: 0.716; 95% CI: 0.559-0.918; *P* = .008), and absence of PVT (HR: 0.718; 95% CI: 0.568-0.909; *P* = .006). Additionally, no conspicuous interaction was noted among diverse groups (all *P *for interaction >.05).

## Discussion

Given the risk of bleeding, anticoagulation needs to be used with extreme caution in cirrhotic patients. Specifically, it remains controversial whether anticoagulation improves the prognosis of cirrhosis, although it is only considered to prevent progressive thrombosis in cirrhotic patients with symptomatic PVT^16^ and, indeed, has been shown to increase thrombus resolution rates. For instance, Chen et al^17^ conducted a retrospective study involving 66 patients with cirrhosis complicated by PVT and observed the limited impact of anticoagulation with warfarin on hepatic decompensation and survival of patients. Another study, which included 80 patients, also elucidated that anticoagulation did not improve overall orthotopic liver transplantation-free survival.^18^ Conversely, a retrospective study found an association between anticoagulant therapy and improved outcomes in cirrhotic patients with PVT, as evidenced by the observation that anticoagulation was associated not only with remarkably lower in-hospital mortality rates but also with reduced incidence of complications such as hepatorenal syndrome, spontaneous bacterial peritonitis, and acute kidney injury.^19^ Similar results were also reported by Villa et al,^20^ who observed that anticoagulation improved survival and delayed the occurrence of hepatic decompensation in outpatients with cirrhosis. In addition, a recent meta-analysis involving 500 patients from 5 studies, none of whom were in the ICU, showed that anticoagulation was associated with reduced all-cause mortality in patients with non-tumoral PVT, independent of thrombosis severity and recanalization.^21^

In the present study, the focus was specifically on patients with liver cirrhosis who were admitted to the ICU, distinguishing this research from previous studies that often included a broader patient population in less critical care settings.^19^^-^^21^ This critical distinction is vital, as ICU patients typically exhibit more severe disease manifestations, comorbid conditions, and complex treatment requirements. The results revealed that early UFH use was associated with lower 30-day mortality in ICU patients with cirrhosis. More importantly, such benefit of UFH treatment was also found in patients without PVT through stratified analyses, highlighting that the protective effect of early UFH use against cirrhosis cannot be simply attributed to recanalization of PVT. While several studies have documented the potential survival benefit of treating PVT in cirrhotic patients, these findings suggest that UFH may provide additional advantages beyond its impact on PVT. The survival benefit of UFH treatment for cirrhotic patients may involve multifactorial mechanistic factors because of the complex pharmacological effects of UFH. As an anticoagulant, UFH can markedly improve hepatic microcirculation and repress sinusoidal capillarization by combating macro- and microvascular thrombosis, further improving liver function and relieving portal hypertension.^8^ Moreover, UFH exerts direct antifibrotic effects both in vivo and in vitro.^22^ Unfractionated heparin can suppress thrombin/factor Xa signals to block the differentiation of hepatic stellate cells into myofibroblasts, which are implicated in liver injury and promote fibrosis.^22^ Additionally, UFH can depress activation of various inflammatory cells and subsequent propagation of inflammatory responses via nuclear factor kappa B(NF-κB) and complement pathways.^23^^,^^24^^,^^25^ These findings prompt the assumption that the anticoagulant, anti-inflammatory, and antifibrotic effects of UFH may account for the potential benefit of early UFH use in improving prognosis in cirrhotic patients. Additionally, through dose stratification analysis, it was found that the low-dose group (≤5000 IU) showed a significant association with reduced 30-day mortality rate. However, no significant association with reduced mortality was observed in the higher dose group (>5000 IU). This result may be attributed to a limited sample size and the speculation that higher doses of UFH increase the risk of adverse events, such as bleeding, potentially obscuring any obscuring any therapeutic benefits.

In cirrhotic patients, bleeding is the most important and common adverse effect to consider before initiating anticoagulation. In this regard, the majority of studies exhibited no correlation between anticoagulant treatment and bleeding complications,^26^^,^^27^^,^^28^^,^^29^ and several studies reported that the risk of bleeding even decreased in patients receiving anticoagulants.^16^^,^^30^ Fuentes et al noticed that cirrhotic patients receiving UFH displayed higher incidence rates of blood product transfusion compared to patients without evidence of cirrhosis and anticoagulated with UFH.^31^ Two other studies elaborated that cirrhotic patients receiving UFH had higher risk of bleeding than those receiving LMWH.^32^^,^^33^ In a study by Drolz et al,^34^ 211 hospitalized patients with cirrhosis in the ICU were examined, and 35 of them experienced severe bleeding during their stay. The author compared the use of heparin between the bleeding group (35 cases) and the non-bleeding group (176 cases) and found no difference in the percentage of heparin use between the 2 groups (6 out of 35 [17%] vs. 32 out of 176 [18%]; *P* = .884). The findings of the study suggested that heparin anticoagulation therapy does not increase the risk of bleeding in ICU patients with cirrhosis. While this study was unable to directly assess bleeding risk due to a lack of precise bleeding timing data in the MIMIC-IV database, RBC transfusion rates given ≥48 hours after ICU admission as a surrogate marker were analyzed. The results demonstrated no significant association between early UFH use and increased transfusion requirements, suggesting that UFH may not substantially increase the risk of clinically significant bleeding in cirrhotic patients. However, this interpretation requires caution for several reasons: First, the control group had significantly lower baseline hemoglobin levels, which likely contributed to their higher transfusion rates independent of anticoagulation status. Second, transfusion-based assessment may miss non-transfused bleeding events. While these findings tentatively suggest that UFH may be reasonably well tolerated in cirrhosis, the exact bleeding risk profile requires confirmation through prospective studies with standardized bleeding assessments.

The higher prevalence of variceal bleeding and UGIB in the control group warrants specific consideration. This pattern likely reflects inherent clinical decision-making rather than a paradoxical treatment effect. Patients with active bleeding or recent bleeding history at admission constitute a high-risk subgroup in whom therapeutic heparin is typically contraindicated due to concerns over exacerbating hemorrhage. Consequently, such patients would be systematically allocated to the non-heparin (control) group. The data source did not permit definitive distinction between bleeding events present at admission (confounders) and those occurring during hospitalization (outcomes). Therefore, these diagnoses were intentionally excluded as covariates in PSM and Cox regression models to avoid introducing immortal time bias or misclassifying baseline confounders as outcomes. The concentration of bleeding diagnoses in the control group aligns with expected real-world practice—where clinicians selectively withhold anticoagulation in bleed-prone patients—and supports the internal validity of the cohort allocation.

Other serious adverse reactions of UFH include hyperkalemia and rare cases of anaphylactic shock. The diagnostic codes available in the database were reviewed, and no cases of UFH-induced thrombocytopenia, hyperkalemia, or anaphylactic shock were identified in the present study’s cohort. Nevertheless, the retrospective nature of the study and the limitations of the database may lead to an underestimation of such events. Future prospective studies with detailed adverse event monitoring are recommended to more accurately assess the safety profile of UFH in this patient population. Additionally, upon searching the dataset, it was found that among the 781 patients in the UFH group, 26 patients received UFH for therapeutic anticoagulation, while the remaining received it for prophylactic purposes. These findings are consistent with established clinical practices in the ICU, where UFH is commonly administered either for prophylaxis or for the treatment of thromboembolic conditions such as deep vein thrombosis, pulmonary embolism, or PVT. Although each treatment cannot be precisely linked to a specific diagnosis, such as acute PVT, due to limitations in diagnostic coding granularity, the distribution of UFH indications in this cohort supports its use based on clinical necessity rather than for study purposes alone.

This study had several strengths. First, this research was the first to probe the correlation of early heparin use with the prognosis in cirrhotic patients. Second, the large sample size in this study strengthened the statistical credibility and reliability. Third, different from most previous trials that only included patients with PVT, this research included a substantial proportion of patients without PVT and demonstrated the survival benefit of UFH for such patients, which reinforced the concept that anticoagulation should be considered as early as possible, rather than after the development of PVT.

The study had several limitations. First, as previously noted, the MIMIC-IV database lacks precise timing data for bleeding events, making it difficult to establish causality between heparin administration and subsequent bleeding episodes. While post–48-hour transfusion requirements were used as a proxy for major bleeding risk associated with heparin use, this methodology has important limitations. Specifically, it may underestimate true bleeding incidence by failing to capture both delayed bleeding events and non-massive bleeding that did not require transfusion. Hence, caution should be exercised in interpreting the results. Second, as a retrospective study using the MIMIC-IV database, this analysis may be subject to unmeasured confounding. A notable limitation is the baseline imbalance in MELD-Na scores (higher in controls) and albumin administration (more frequent in the UFH group), which suggests potential treatment selection bias. While PSM was employed to mitigate known confounders, residual confounding may persist due to unmeasured clinical factors influencing treatment decisions. Third, given the single-center retrospective observational design of this research, randomized controlled trials are required to further validate the results. Fourth, LMWH is a preferred choice of heparin therapy. However, it was found that the sample size of ICU patients with liver cirrhosis receiving LMWH in the MIMIC-IV database was too small to conduct a meaningful statistical analysis. Future research can further explore the association between LMWH and mortality in cirrhotic patients. Lastly, using data exclusively from the MIMIC-IV database, which comprises patients admitted to the ICU, may limit the generalizability of these findings to a broader population of cirrhosis patients. Future studies should include a more diverse patient population, including both inpatient and outpatient settings, which will help to elucidate the efficacy of UFH across different stages of cirrhosis and varying clinical contexts.

In conclusion, the current study found that early heparin use was associated with a decrease in 30-day mortality in cirrhotic patients, without a significant increase in RBC transfusion. These findings offer a novel insight into the potential efficacy of heparin in the treatment of cirrhosis, thereby further advancing research in this field.

## Figures and Tables

**Table 1.** Comparison of Baseline Characteristics Between the Early Unfractionated Heparin Group and the Control Group

**Variables**	**Control Group**	**Early UFH group**	***P***
N	871	781	
Sex, n (%)			.375
Female	299 (34.33)	252 (32.27)	
Male	572 (65.67)	529 (67.73)	
Age (years), mean (SD)	58.12 (11.81)	59.60 (12.00)	.012
Albumin(g/dL), mean (SD)	2.97 (0.66)	2.90 (0.64)	.027
ALT (IU/L), median (Q1, Q3)	33.00 (21.00-53.00)	35.45 (20.00-63.00)	.150
AST (IU/L), median (Q1, Q3)	67.00 (40.00-119.00)	63.00 (37.00-119.95)	.281
Platelets (K/uL), median (Q1, Q3)	92.00 (57.00-141.50)	112.00 (76.00-166.00)	<.001
Sodium (mEq/L), mean (SD)	135.71 (5.78)	134.54 (6.68)	<.001
Creatinine (mg/dL)	1.00 (0.70-1.30)	1.00 (0.70-1.50)	.149
Hemoglobin (g/dL), mean (SD)	9.28 (1.99)	10.18 (2.10)	<.001
Chloride (mEq/L), mean (SD)	102.59 (6.79)	101.73 (7.24)	.012
Bilirubin (mg/dL), median (Q1, Q3)	2.90 (1.40-7.25)	2.40 (1.20-5.20)	<.001
INR, median (Q1, Q3)	1.80 (0.63)	1.69 (0.57)	<.001
Magnesium (mg/dL), mean (SD)	1.89 (0.38)	1.88 (0.37)	.578
Phosphorus (mg/dL), mean (SD)	3.61 (1.20)	3.60 (1.29)	.870
PTT (s), mean (SD)	38.91 (11.63)	38.51 (11.64)	.485
Calcium (mg/dL), mean (SD)	8.16 (0.79)	8.27 (0.83)	.006
WBC (K/uL), median (Q1, Q3)	8.70 (5.60-12.70)	9.60 (6.30-14.20)	.003
Alcoholic cirrhosis, n (%)			<.001
No	184 (21.13)	245 (31.37)	
Yes	687 (78.87)	536 (68.63)	
Renal failure, n (%)			.382
No	760 (87.26)	670 (85.79)	
Yes	111 (12.74)	111 (14.21)	
Chronic pulmonary disease, n (%)			.389
No	780 (89.55)	689 (88.22)	
Yes	91 (10.45)	92 (11.78)	
Heart failure, n (%)			.155
No	778 (89.32)	680 (87.07)	
Yes	93 (10.68)	101 (12.93)	
Diabetes, n (%)			.378
No	808 (92.77)	733 (93.85)	
Yes	63 (7.23)	48 (6.15)	
Sepsis, n (%)			.743
No	290 (33.30)	266 (34.06)	
Yes	581 (66.70)	515 (65.94)	
Liver cancer, n (%)			.659
No	823 (94.49)	734 (93.98)	
Yes	48 (5.51)	47 (6.02)	
VTE, n (%)			.268
No	848 (97.36)	753 (96.41)	
Yes	23 (2.64)	28 (3.59)	
HE, n (%)			.017
No	748 (85.88)	701 (89.76)	
Yes	123 (14.12)	80 (10.24)	
PVT, n (%)			.787
No	830 (95.29)	742 (95.01)	
Yes	41 (4.71)	39 (4.99)	
MELD score mean (SD)	20.77 (9.06)	19.57 (8.76)	.006
SOFA score, median (Q1, Q3)	7.48 (3.76)	7.10 (3.54)	.037
Mechanical ventilation, n (%)			.081
No	503 (57.75)	484 (61.97)	
Yes	368 (42.25)	297 (38.03)	
Diuretic use, n (%)			.858
No	639 (73.36)	576 (73.75)	
Yes	232 (26.64)	205 (26.25)	
Vasopressor use, n (%)			.310
No	597 (68.54)	517 (66.20)	
Yes	274 (31.46)	264 (33.803)	
Albumin use, n (%)	0.00 (0.00-50.00)	0.00 (0.00-50.00)	.01
No	546 (62.69)	441 (56.47)	
Yes	325 (37.31)	340 (43.53)	
UGIB, n (%)			<.001
No	622 (71.412)	732 (93.726)	
Yes	249 (28.588)	49 (6.274)	
Variceal bleeding, n (%)			<.001
No	689 (79.104)	748 (95.775)	
Yes	182 (20.896)	33 (4.225)	
Mortality, n (%)			.007
No	654 (75.09)	630 (80.67)	
Yes	217 (24.91)	151 (19.33)	
Transfusion, n (%)			.052
No	696 (79.91)	653 (83.61)	
Yes	175 (20.09)	128 (16.39)	

ALT, alanine transaminase; AST, aspartate transaminase; HE, hepatic encephalopathy; INR, international normalized ratio; MELD, model for end-stage liver disease; PTT, partial thromboplastin time; PVT, portal venous thrombosis; SOFA, sequential organ failure assessment; UFH, unfractionated heparin; UGIB, upper gastrointestinal bleeding; VTE, venous thromboembolism; WBC, white blood cell.

**Table 2.** Comparison of the Baseline Characteristics After Propensity Score Matching

**Variables**	**Control Group**	Early UFH group	***P***
N	579	579	
Sex, n (%)			.664
Female	199 (34.37)	192 (33.16)	
Male	380 (65.63)	387 (66.84)	
Age (years), mean (SD)	58.56 (11.90)	58.62 (11.98)	.928
Albumin(g/dL), mean (SD)	2.91 (0.66)	2.90 (0.65)	.841
ALT (IU/L), median [Q1, Q3]	33.00 (21.00-55.00)	33.00 (20.00-61.00)	.943
AST(IU/L), median [Q1, Q3]	66.00 (39.00-120.00)	64.00 (38.00-116.25)	.591
Platelets (K/uL), median [Q1, Q3]	101.00 (62.50-160.50)	106.00 (68.00-152.00)	.218
Sodium (mEq/L), mean (SD)	135.17 (5.90)	135.02 (6.44)	.690
Creatinine (mg/dL), median [Q1, Q3]	1.00 (0.70-1.30)	1.00 (0.70-1.40)	.413
Hemoglobin (g/dL), mean (SD)	9.82 (1.99)	9.70 (1.91)	.300
Chloride (mEq/L), mean (SD)	102.18 (6.71)	102.21 (7.13)	.957
Bilirubin (mg/dL), median [Q1, Q3]	2.70 (1.30-6.60)	2.70 (1.30-5.95)	.500
INR, median [Q1, Q3]	1.60 (1.30-1.95)	1.60 (1.30-2.00)	.702
Magnesium (mg/dL), mean (SD)	1.90 (0.38)	1.87 (0.37)	.182
Phosphorus (mg/dL), mean (SD)	3.61 (1.25) 3.50 (2.80-4.10)	3.56 (1.30) 3.40 (2.70-4.10)	.472
PTT (s), mean (SD)	38.45 (11.43)	38.87 (11.95)	.544
Calcium (mg/dL), mean (SD)	8.18 (0.80)	8.19 (0.79)	.813
WBC (K/uL), median (Q1, Q3)	8.90 (5.70-13.15)	9.10 (6.00-13.50)	.662
Alcoholic cirrhosis, n (%)			.840
No	149 (25.73)	146 (25.22)	
Yes	430 (74.27)	433 (74.78)	
Renal failure, n (%)			.243
No	515 (88.95)	502 (86.70)	
Yes	64 (11.05)	77 (13.30)	
Chronic pulmonary disease, n (%)			.782
No	514 (88.77)	511 (88.26)	
Yes	65 (11.23)	68 (11.74)	
Heart failure, n (%)			.928
No	510 (88.08)	509 (87.91)	
Yes	69 (11.92)	70 (12.09)	
Diabetes, n (%)			.904
No	542 (93.61)	543 (93.78)	
Yes	37 (6.39)	36 (6.22)	
Sepsis, n (%)			.708
No	194 (33.51)	188 (32.47)	
Yes	385 (66.49)	391 (67.53)	
Liver cancer, n (%)			.391
No	540 (93.26)	547 (94.47)	
Yes	39 (6.74)	32 (5.53)	
HE, n (%)			.786
No	508 (87.74)	511 (88.26)	
Yes	71 (12.26)	68 (11.74)	
PVT, n (%)			.777
No	552 (95.34)	554 (95.68)	
Yes	27 (4.66)	25 (4.32)	
MELD score, mean (SD)	20.10 (8.85)	20.14 (8.77)	.931
SOFA score, median (Q1, Q3)	7.00 (4.00-10.00)	7.00 (5.00-9.00)	.547
Mechanical ventilation, n (%)			.588
No	357 (61.66)	348 (60.10)	
Yes	222 (38.34)	231 (39.90)	
Diuretic use, n (%)			.739
No	423 (73.06)	428 (73.92)	
Yes	156 (26.94)	151 (26.08)	
Vasopressor use, n (%)			.616
No	387 (66.84)	395 (68.22)	
Yes	192 (33.16)	184 (31.78)	
Albumin use (g), n (%)			.591
No	335 (57.86)	344 (59.41)	
Yes	244 (42.14)	235 (40.59)	
UGIB, n (%)			<.001
No	427 (73.75)	535 (92.40)	
Yes	152 (26.25)	44 (7.60)	
Variceal bleeding, n (%)			<.001
No	466 (80.48)	549 (94.82)	
Yes	113 (19.52)	30 (5.18)	
Mortality, n (%)			.018
No	438 (75.65)	471 (81.35)	
Yes	141 (24.35)	108 (18.65)	
Transfusion, n (%)			.440
No	472 (81.52)	482 (83.25)	
Yes	107 (18.48)	97 (16.75)	

ALT, alanine transaminase; AST, aspartate transaminase; HE, hepatic encephalopathy; INR, international normalized ratio; MELD, model for end-stage liver disease; PTT, partial thromboplastin time; PVT, portal venous thrombosis; SOFA, sequential organ failure assessment; UFH, unfractionated heparin; UGIB, upper gastrointestinal bleeding; WBC, white blood cell.

**Table 3.** Association Between Unfractionated Heparin Use and 30-day Mortality Using Different Models

**Model**	**HR (95% CI) **	*P*
Cox proportional hazards models		
Model I*	0.744 (0.605-0.917)	.005
Model II**	0.770 (0.626-0.949)	.014
Model III***	0.753 (0.609-0.930)	.008
Model IIII****	0.742 (0.599-0.919)	.006
Propensity score matching model	0.729 (0.568-0.937)	.014

*Non-adjusted.

**Adjusted for age, model for end-stage liver disease score, and sequential organ failure assessment score.

***Adjusted for age, model for end-stage liver disease score, sequential organ failure assessment score, international normalized ratio, albumin, albumin use, and sodium.

****Age, model for end-stage liver disease score, sequential organ failure assessment score, international normalized ratio, albumin, albumin use, sodium, alcoholic cirrhosis, chloride, calcium, hemoglobin, platelet, white blood cell, bilirubin, hepatic encephalopathy, and mechanical ventilation.

**Table 4.** Association Between Different Dosages of Unfractionated Heparin Use and 30-day Mortality

**48 Hours Dose of UFH**	**Number**	**OR (95% CI) **	P*
Control group	871	Ref	
≤5000 IU	606	0.766 (0.608, 0.965)	.024
>5000 IU	175	0.712(0.498,1.016)	.061

OR, odds ratio; UFH, unfractionated heparin.

*Adjusted for age, model for end-stage liver disease score, sequential organ failure assessment score, international normalized ratio, albumin, albumin use, sodium, alcoholic cirrhosis, chloride, calcium, hemoglobin, platelet, white blood cell, bilirubin, hepatic encephalopathy, and mechanical ventilation.

**Table 5.** Association Between Unfractionated Heparin Use and 30-Day Mortality in Various Subgroups

Subgroup*	**Number**	**HR (95% CI)**	*P*	***P* for Interaction**
MELD score				.0624
≤20	921	0.994 (0.647-1.527)	.977	
>20	731	0.660 (0.505-0.862)	.002	
Sepsis				.136
No	556	0.898 (0.541-1.491)	.678	
Yes	1096	0.675 (0.522-0.874)	.003	
HE				.791
No	1449	0.716 (0.559-0.918)	.008	
Yes	203	0.509 (0.263-0.983)	.044	
PVT				.905
No	1572	0.718 (0.568-0.909)	.006	
Yes	80	0.95 (0.134-6.748)	.961	
SOFA score				.413
<8	921	0.819 (0.548-1.224)	.330	
≥8	731	0.667 (0.507-0.878)	.004	

HE, hepatic encephalopathy; HR, hazard ratio; MELD, model for end-stage liver disease; PVT, portal venous thrombosis; SOFA, sequential organ failure assessment.

*Adjusted for age, MELD score, SOFA score, international normalized ratio, albumin, albumin use, sodium, alcoholic cirrhosis, chloride, calcium, hemoglobin, platelet, WBC, bilirubin, hepatic encephalopathy, and mechanical ventilation, except for the stratifying variable.

## Data Availability

The datasets analyzed during the current study are available in the physionet (https://physionet.org/content/mimiciv/0.4/).
